# Heterologous prime-boost vaccination with H3N2 influenza viruses of swine favors cross-clade antibody responses and protection

**DOI:** 10.1038/s41541-017-0012-x

**Published:** 2017-04-20

**Authors:** Kristien Van Reeth, José Carlos Mancera Gracia, Ivan Trus, Lieve Sys, Gerwin Claes, Han Versnaeyen, Eric Cox, Florian Krammer, Yu Qiu

**Affiliations:** 1 Laboratory of Virology, Faculty of Veterinary Medicine, Ghent University, Gent, Belgium; 2 Laboratory of Pathology, Faculty of Veterinary Medicine, Ghent University, Gent, Belgium; 3 Laboratory of Immunology, Faculty of Veterinary Medicine, Ghent University, Gent, Belgium; 4 Department of Microbiology, Icahn School of Medicine at Mount Sinai, New York, NY USA; 5 OIE Sub-Regional Representation for South-East Asia, Bangkok, Thailand

## Abstract

The emergence of multiple novel lineages of H1 and H3 influenza A viruses in swine has confounded control by inactivated vaccines. Because of substantial genetic and geographic heterogeneity among circulating swine influenza viruses, one vaccine strain per subtype cannot be efficacious against all of the current lineages. We have performed vaccination-challenge studies in pigs to examine whether priming and booster vaccinations with antigenically distinct H3N2 swine influenza viruses could broaden antibody responses and protection. We prepared monovalent whole inactivated, adjuvanted vaccines based on a European and a North American H3N2 swine influenza virus, which showed 81.5% aa homology in the HA1 region of the hemagglutinin and 83.4% in the neuraminidase. Our data show that (i) Priming with European and boosting with North American H3N2 swine influenza virus induces antibodies and protection against both vaccine strains, unlike prime-boost vaccination with a single virus or a single administration of bivalent vaccine. (ii) The heterologous prime-boost vaccination enhances hemagglutination inhibiting, virus neutralizing and neuraminidase inhibiting antibody responses against H3N2 viruses that are antigenically distinct from both vaccine strains. Antibody titers to the most divergent viruses were higher than after two administrations of bivalent vaccine. (iii) However, it does not induce antibodies to the conserved hemagglutinin stalk or to other hemagglutinin subtypes. We conclude that heterologous prime-boost vaccination might broaden protection to H3N2 swine influenza viruses and reduce the total amount of vaccine needed. This strategy holds potential for vaccination against influenza viruses from both humans and swine and for a better control of (reverse) zoonotic transmission of influenza viruses.

## Influenza: More efficient, more protective vaccinations

Boosting an influenza vaccine with a different strain offers twice the protection for half the volume of vaccine. Pigs and humans need two doses of killed influenza vaccine—a “priming” dose, with a “booster” given approximately 4 weeks after—in order to provide immunity. Kristien Van Reeth, of Belgium’s Ghent University, and collaborators demonstrated that differing the strain of the primer and booster elicits antibodies that protect against both strains, negating the need for two doses of both strains. The swine vaccinated in Van Reeth’s study also had increased immunity against strains that the animals were not vaccinated against but bore similarities to the viruses used. This cross-protection corresponds with conclusions from similar research into other animals and could eliminate the need for unnecessary vaccinations whilst increasing vaccine efficacy in swine and presumably in humans.

## Introduction

Inactivated influenza vaccines protect by inducing serum antibody against the hemagglutinin (HA) and, to a lesser degree, the neuraminidase (NA) of the vaccine strains. While the HA is highly immunogenic and the target for neutralizing antibodies, it is also the most variable protein. For this reason, human influenza vaccine strains are updated every few years.^1^ Influenza A viruses of H1N1, H3N2, and H1N2 subtypes are enzootic in swine worldwide^2^ and both the viruses and the inactivated vaccines for swine resemble those of humans. However, while the human vaccines generally contain purified viral surface antigens without adjuvant, swine influenza virus (SIV) vaccines are mostly whole virus preparations with an oil-based adjuvant. Also, there is no formal system to recommend vaccine strains for swine and SIV vaccine strains are only rarely updated.^3^ This is in part due to slower antigenic drift of influenza viruses in swine than in humans^4, 5^ and to regulatory and economic constraints. Some commercial oil-adjuvanted SIV vaccines have been shown to protect against SIVs isolated decades after the introduction of the vaccine and with considerable antigenic changes.^3, 6^ On the other hand, multiple novel antigenically distinct H1 and H3 SIV lineages have emerged during the last 20 years^2, 5, 7^ and they are now co-circulating in many regions. The prevailing lineages also differ between continents and regions, and SIV vaccines for Europe and North America are produced locally and they contain completely different strains.^3^ Thus, it is unlikely that one strain per subtype would be efficacious globally or within a given region.

All known H3N2 SIVs have HA and NA proteins derived from viruses that once circulated in the human population.^2, 8^ Shortly after the Hong Kong influenza pandemic in 1968, human-like H3N2 influenza viruses were isolated from European pigs.^2^ In the US swine population, three separate introductions of A/Nanchang/95-like human H3N2 viruses occurred between 1995 and 1997, leading to three antigenic clusters (I, II, III) in swine.^2^ The now dominant cluster IV has evolved from cluster III around 2005^9^ and is mostly found with a more recent human N2 from around 2002, though some viruses still have the original N2 from 1995 strains.^10^ On both continents, the internal genes of the human seasonal H3N2 viruses have been replaced with those of viruses that were likely better adapted for replication in swine by reassortment. This has led to the designation “triple-reassortant” SIV in North America, because of a fixed combination of internal genes of human, avian, and swine virus origin.^9^ More recently the 2009 pandemic H1N1 virus has reassorted extensively with the previously established SIVs.^7, 10^ One of many novel genotypes, a triple reassortant H3N2 SIV in which only the matrix gene comes from the 2009 pandemic virus, has infected over 350 humans since 2011.^9^ This virus is also known as H3N2 “variant” or H3N2“v”, a designation used for SIVs that are isolated from humans. Since 2012, H3N2 and H3N1 viruses with an HA from human strains from the 2010–2011 season have also been reported in swine in the US.^11^ Thus, the frequent introduction of human H3N2 viruses and the geographic separation of the swine population have led to considerable diversity in SIVs in different regions. Swine H3N2 viruses are also no longer similar to the current human H3N2 viruses because of a different evolution in swine vs. humans. Swine can act as reservoirs for older human HAs and several studies have shown a lack of protective serum antibody titers against H3N2 SIVs in people born after the circulation of the respective human precursor viruses.^12–14^ SIVs therefore pose a threat for reintroduction into the human population once immunity has waned sufficiently to allow widespread infection.

Influenza naïve humans or pigs need two doses of killed influenza vaccine, 3 or 4 weeks apart, to achieve a satisfactory level of antibody on primary immunization. One approach to broaden the immune response is to use antigens from divergent virus strains for primary and booster vaccinations. This has been documented in vaccination experiments with avian H5N1 influenza viruses in poultry^15^ and in humans,^16, 17^ and with human H1N1 and H3N2 viruses in the mouse and ferret model.^18–20^ In 2009, vaccination with the novel pandemic H1N1 virus resulted in a broad, pan-H1 neutralizing antibody response in people who had been exposed to human seasonal H1N1 viruses from 1977–2008, despite a lack of serological cross-reactivity between these H1N1 viruses.^21^ Here we aimed to examine whether primary and booster vaccinations with antigenically distinct European and North American H3N2 SIVs could broaden antibody responses and protection in swine.

## Results

### Heterologous prime-boost vaccination with European and North American H3N2 SIVs induces antibody titers against both vaccine strains

We prepared adjuvanted inactivated, monovalent whole virus vaccines based on contemporary European (sw/Gent/172/2008, G08) and North American (sw/Pennsylvania/A01076777/2010, PA10) H3N2 SIVs. These strains show low amino acid (aa) homology in the HA1 subunit (81.5%) of the HA and in the NA (83.4%), and as many as 20 aa differences at putative antigenic sites of the HA1 (Figs. S1,2) and NA on a total of 40 and 34 aa residues, respectively.^11, 22–24^

Pigs were injected first with G08 vaccine and boosted 4 weeks later with PA10 vaccine (heterologous prime-boost group) or primed and boosted with identical virus strains, either G08 or PA10 (homologous prime-boost groups). We also included pigs that received a single (bivalent vaccine 1x group) or two consecutive administrations (bivalent vaccine 2x group) of a combination vaccine containing both strains, or two administrations of mock vaccine.

**Fig. 1:**
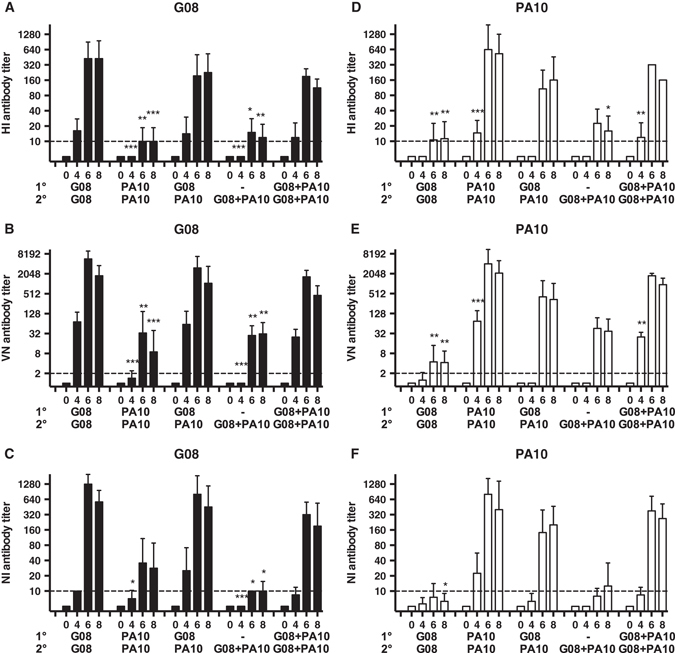
Heterologous prime-boost vaccination with G08 and PA10 induces antibodies against both vaccine strains. Serum antibody titers are compared with those achieved by homologous prime-boost vaccination and one or two administrations of bivalent vaccine. The time interval between the priming (1°) and booster (2°) immunizations was 4 weeks. HI (**a**, **d**), VN (**b**, **e**) and NI (**c**, **f**) antibody titers were determined at 4 timepoints: before the priming vaccination, 4 weeks after the priming vaccination and before the booster vaccination, and 2 and 4 weeks after the booster vaccination (weeks 0, 4, 6, and 8). The vaccine groups are grouped along the x-axis. The bivalent vaccine (1x) group received a single vaccine administration at week 4 of the experiment. Geometric mean antibody titers against G08 and PA10 are shown in figures **a**–**c** and **d**–**f** respectively. Error bars represent SD. Dotted lines indicate limits of detection. Pigs of the mock-vaccinated control group (not shown) had no detectable antibody titers in all three assays. Asterisks denote significant differences between the heterologous prime-boost and other groups in the Kruskal-Wallis test. *P < 0.05; **P < 0.01; ***P < 0.001

We first examined the evolution of antibody titers against both vaccine strains by hemagglutination inhibition (HI), virus neutralization (VN) and neuraminidase inhibition (NI). Sera from mock-vaccinated control pigs and prevaccination sera tested negative in all three assays. Postvaccination antibody titers were higher in the VN than in HI or NI assays, but the three assays revealed a similar pattern (Fig. 1a–f). After a single administration of monovalent or bivalent vaccine most pigs had minimal or low antibody titers against the virus(es) in the vaccine. Data at week 6 and 8 demonstrate the effect of the booster vaccination. Priming and boosting with identical virus strains induced high antibody titers against the vaccine strain only, which were comparable in both groups, but minimal titers to the heterologous virus. The heterologous prime-boost group, in contrast, mounted solid HI, VN, and NI antibody titers against both vaccine strains, which did not differ significantly from those in the homologous prime-boost groups (*P* *≥* 0.3416). Anti-G08 titers were significantly higher in the heterologous prime-boost group than in the bivalent vaccine (1x) group, but there were no significant differences in anti-PA10 titers, except for HI titers at week 8. This can be explained by the higher antibody titers against G08 than against PA10 upon the heterologous boost. Importantly, antibody titers against both vaccine strains were comparable in the heterologous prime-boost and bivalent vaccine (2x) groups (*P* *≥* 0.9999) (Table S1).

**Fig. 2:**
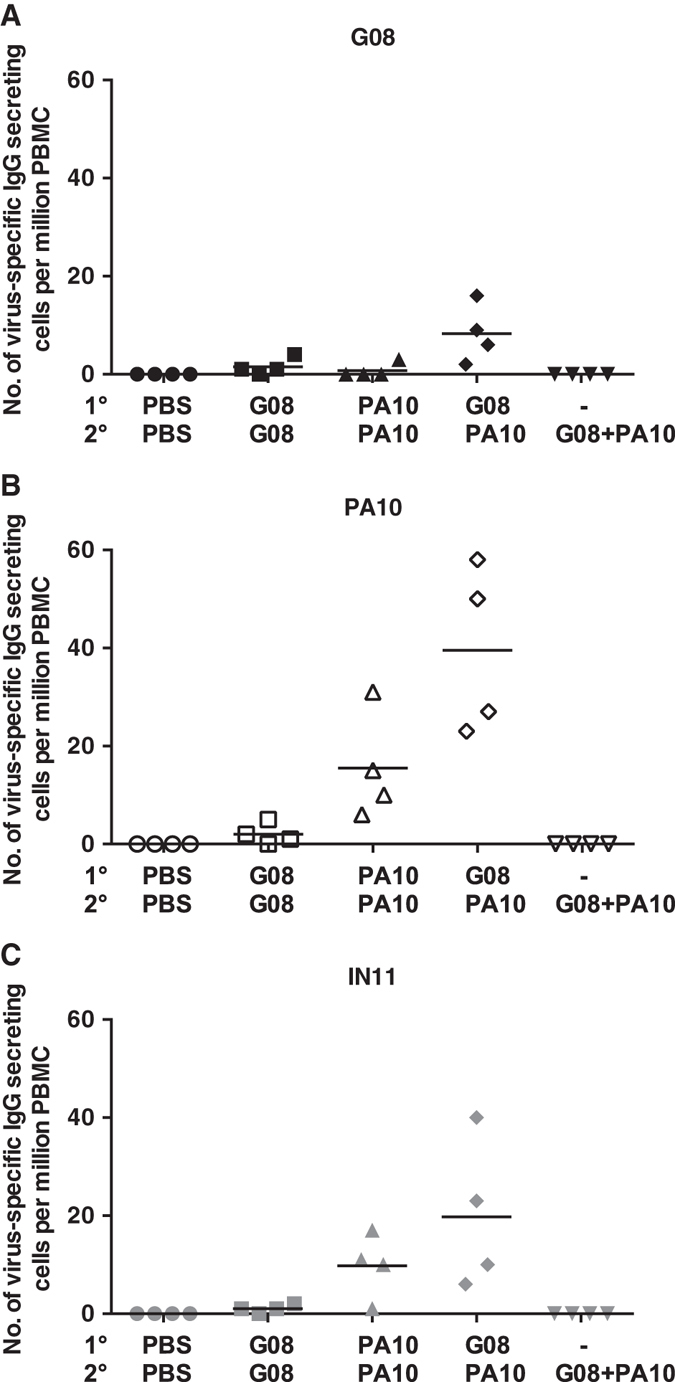
Higher numbers of vaccine-specific antibody secreting cells (ASC) in pigs of the heterologous prime-boost group as compared to homologous prime-boost and bivalent vaccine (1x) groups. Peripheral blood mononuclear cells of four pigs per group were collected at the time of the booster or single vaccination and 7 days later. Numbers of IgG ASC were determined by direct ELISPOT against three viruses: the vaccine strains G08 (**a**) and PA10 (**b**) and the H3N2v virus A/Indiana/08/2011 (IN11, **c**), which is related to PA10. Virus-specific responses were undetectable at the time of vaccination, results 7 days postvaccination are shown. Each data point represents an individual pig, horizontal lines indicate group means. Numbers of virus-specific ASC against all three virus strains were higher in pigs from the heterologous prime-boost group as compared to the mock-vaccinated control and bivalent vaccine (1x) groups (*P* < 0.01, Kruskal-Wallis test), and the G08 and PA10 homologous prime-boost groups (*P* > 0.05)

We also wanted to compare the frequency of vaccine-specific antibody secreting cells (ASC) between groups. To this purpose we collected peripheral blood mononuclear cells (PBMC) from four pigs of each group, except for the bivalent vaccine (2x) group, just before the booster or single vaccination and 7 days later. These timepoints were selected because ASC responses have been shown to peak 1 week after immunization of primed subjects.^21^ Numbers of IgG secreting PBMC were determined in an ELISPOT assay, using either one of both vaccine strains as stimuli, as well as the H3N2v virus A/Indiana/08/2011 (IN11). Virus-specific ASC were undetectable in all pigs at the time of vaccination, and 7 days later in the mock-vaccinated control and bivalent vaccine (1x) groups (Fig. 2a–c). The homologous boost with PA10 resulted in ASC reacting with the vaccine virus and the related IN11 virus (Fig. 2b,c). Both homologous prime-boost groups had minimal responses to G08 (Fig. 2a). The heterologous prime-boost group showed increased numbers of ASC against all three viruses. Two of the four pigs in this group had substantially higher responses than the PA10 homologous prime-boost group. However, the differences between vaccine groups were not statistically significant (*P* > 0.05) because of a large degree of inter-animal variability.

These results suggest that the humoral immunity induced by G08 vaccine primes pigs for stronger responses to both vaccine viruses on subsequent vaccination with PA10.

### Heterologous prime-boost vaccination with G08 and PA10 protects against challenge with either one of both vaccine strains

The next step was to demonstrate the extent of protection against challenge with the vaccine strains. Though SIVs replicate throughout the respiratory tract of pigs, the lungs are the major target and lung virus titers correlate with disease. Yet typical clinical signs can only be reproduced by direct intratracheal inoculation of a high virus dose, which is artificial and results in delayed virus replication in the upper airways.^3, 6^ We therefore chose to inoculate the pigs intranasally and to determine virus titers in the lung, trachea and nasal mucosa.

**Fig. 3:**
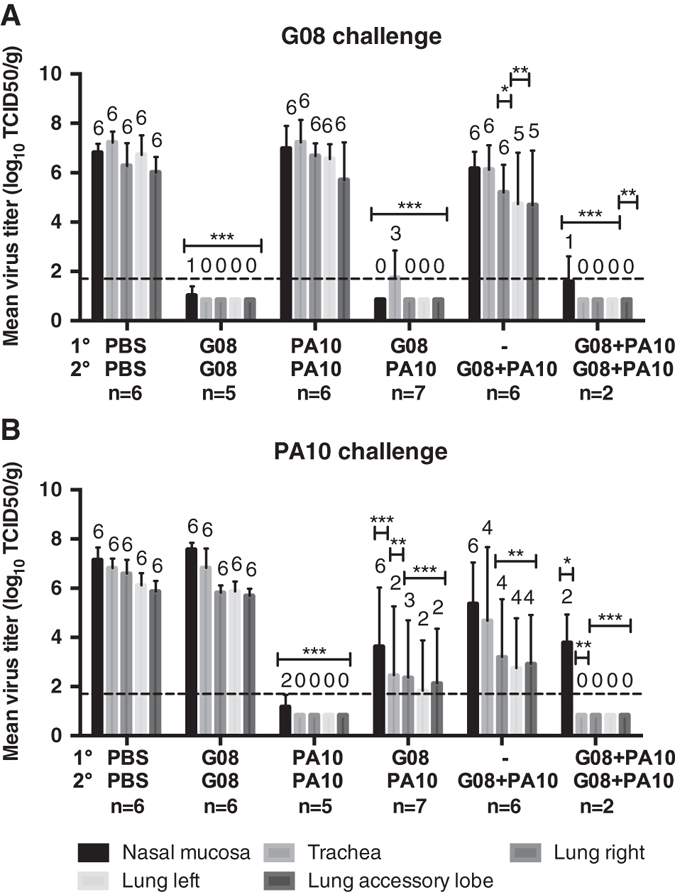
Heterologous prime-boost vaccination with G08 and PA10 protects against challenge with either one of both vaccine strains. Pigs were challenged intranasally with 10^7^ TCID_50_ of G08 (**a**) or PA10 (**b**) 1 month after the booster vaccination or after the single vaccination in the bivalent vaccine (1x) group. They were euthanized 3 days after the challenge, and virus titers were determined in samples of the nasal mucosa, trachea and three different parts of the lung. Bars represent mean virus titers ± SD. Dotted lines indicate the limit of detection. Numbers along the x-axis represent the number of pigs challenged with G08 or PA10 in each group. Numbers on top of the bars indicate the number of pigs with detectable viral titers. Asterisks indicate significantly reduced virus titers as compared to the mock-vaccinated challenge control group in ANOVA. *P < 0.05, **P < 0.01, ***P < 0.001. The normal distribution was assessed by the Shapiro-Wilk test. Statistically significant reductions of the number of virus-positive pigs were observed upon G08 challenge in the G08 homologous prime-boost (*P* < 0.05 for nasal mucosa and *P* < 0.01 for other samples, Fisher’s exact test) and heterologous prime-boost groups (*P* < 0.001, all samples except trachea) and upon PA10 challenge in the PA10 homologous prime-boost (*P* < 0.01, all samples except nasal mucosa) and heterologous prime-boost groups (*P* < 0.05, all samples except nasal mucosa). A significant reduction was also observed in the bivalent vaccine (2x) group for trachea and lung samples (*P* < 0.05)

Both G08 and PA10 replicated to high titers in all samples of the challenge control pigs (Fig. 3a, b). Homologous prime-boost vaccination with G08 or PA10 offered a complete protection against replication of the homologous challenge virus, except for one or two samples of the nasal mucosa. Protection against heterologous challenge, in contrast, was lacking. Heterologous prime-boost vaccination protected completely against challenge with G08, except for three tracheal samples (*P* < 0.01 for all pigs). After challenge with PA10, most pigs did not have detectable virus in the lungs and trachea, and mean virus titers were significantly reduced (*P* < 0.001 for lungs and nasal mucosa, *P* < 0.01 for trachea). One administration of bivalent vaccine resulted in an inferior protection against both viruses. In the bivalent vaccine (2x) pigs virus was recovered from the nasal mucosa only in one (G08) or both pigs (PA10).

Intranasal inoculation of pigs with SIV causes only mild lesions of the respiratory tract. However, enhanced lesions as compared to those in unvaccinated challenge controls had been reported in pigs vaccinated with adjuvanted inactivated vaccine followed by challenge with a homosubtypic but antigenically distinct SIV.^25^ This has prompted us to perform (histo)pathological examinations of the lungs and trachea. Unvaccinated, unchallenged control pigs, which tested negative for SIV and antibodies until the end of the study, did not show gross lung lesions or microscopic lesions of the trachea. Lung epithelial damage and peribronchiolar lymphocytic cuffing were occasionally seen. Only part of the mock-vaccinated challenge control pigs showed macroscopic lung lesions, involving ≤10% of the lungs, after challenge with G08 or PA10 (Table S2). Microscopic lesions were also mild and consisted of accumulations of lymphocytes and neutrophils in or around bronchioli, bronchiolar epithelial damage, thickening of the alveolar walls and focal attenuation of the tracheal epithelium (Table S2, Fig. S3).

The vaccinated pigs also had no or minimal macroscopic pneumonia, and mild microscopic lesions. Compared to the challenge control groups, most vaccinated groups had milder microscopic lesion scores. This was most prominent for the bivalent vaccine (2x) groups and the homologous prime-boost groups challenged with homologous virus, but none of the differences between vaccinated and control groups were statistically significant (*P* = 0.06–1.00, Kruskal-Wallis; *P* = 0.08–1.00, two-sided Fisher’s exact test). We found only moderate correlations between lesion scores and viral loads in the lungs or trachea of individual pigs (Spearman’s rho = 0.37 and 0.59, *P* < 0.01). Most important, none of the vaccine groups showed evidence of enhanced lesions relative to the challenge control.

### Heterologous prime-boost vaccination enhances serological cross-reactivity with H3N2 viruses that are antigenically distinct from both vaccine strains

To further analyse the breadth of the antibody response, we examined sera collected 2 weeks after the booster vaccination for their cross-reactivity with heterologous H3N2 or H1N2 SIVs and human seasonal H3N2 viruses of different antigenic clusters (Tables 1, 2). A/Victoria/3/1975 and A/Nanchang/933/1995 are the supposed human precursors of the European and North American H3N2 SIV lineages respectively. The antigenic distance between H3N2 SIVs in Europe and North America is on average 12.6 antigenic units (AU).^2^ The recent human H3N2 virus A/Victoria/361/2011 is 14.9 AU away from contemporary European H3N2 SIVs and 6.4 AU from North American H3N2 SIVs. H3N2 SIVs show lower antigenic diversity in Europe (0.28 AU per year) than in the US (0.49 AU per year). Though A/Indiana/08/2011, an H3N2v virus, and sw/Iowa/A01049750/2011 are cluster IV North American H3N2 viruses, they are considered antigenically distant from PA10.^4^ The average distance between consecutive human clusters is 4.5 AU.^26^ Human influenza vaccine strains are updated when there is an antigenic difference of at least 2 units between the vaccine strain and the strains expected to circulate in the next season.

**Table 1:** Influenza virus strains used in serological assays and their genetic relationship with the HA and NA of both vaccine strains

				% amino acid identity to					
		Origin of		sw/Gent/2008 (G08)			sw/PA/2010 (PA10)		
Virus lineage	Virus strain	HA gene	NA gene	HA1	HA2	NA	HA1	HA2	NA
Eu swine H3N2	Sw/Gent/1984	hu ‘73–‘75	hu ‘73–‘75	89.4 (7)*	92.3	93.7 (4)	80.5 (23)	93.2	86.5 (19)
	Sw/Eng/1987	hu ‘73–‘75	hu ‘73–‘75	83.0 (11)	88.7	89.0 (7)	79.6 (20)	91.9	86.8 (17)
	Sw/Gent/2008	hu ‘73–‘75	hu ‘73–‘75	100	100	100	81.5 (20)	91.0	83.4 (20)
Eu swine H1N2	Sw/Gent/2012	hu ‘80	hu ‘73–‘75	n.a.	n.a.	87.0 (10)	n.a.	n.a.	83.8 (15)
N A swine H3N2 cluster I	Sw/TX/1998	hu ‘95–‘96	hu ‘95–‘96	82.4 (19)	92.3	87.9 (14)	88.8 (13)	95.0	91.7 (11)
	Sw/MN/1999	hu ‘95–‘96	hu ‘95–‘96	82.1 (20)	92.3	88.1 (13)	88.8 (13)	95.0	91.5 (12)
N A swine H3N2 cluster IV	Sw/ON/2005	hu ‘95–‘96	hu ‘95–‘96	81.5 (19)	91.9	85.6 (16)	96.7 (5)	98.2	95.7 (10)
	Sw/PA/2010	hu ‘95–‘96	hu ‘01–‘02	81.5 (20)	91.0	83.4 (20)	100	100	100
	A/IN/2011	hu ‘95–‘96	hu ‘01–‘02	79.6 (18)	90.0	83.2 (20)	94.2 (4)	95.9	99.8 (0)
	Sw/IA/2011	hu ‘95–‘96	hu ‘95–‘96	80.2 (17)	90.0	88.8 (11)	93.6 (6)	95.5	89.1 (14)
Human seasonal H3N2	A/Vict/1975	n.a.	n.a.	86.6 (11)	92.3	90.9 (9)	83.6 (22)	95.0	88.7 (15)
	A/Eng/1988	n.a.	n.a.	85.1 (13)	92.3	89.4 (12)	90.0 (13)	95.0	90.4 (13)
	A/Nanch/1995	n.a.	n.a.	83.0 (17)	92.8	88.1 (13)	91.5 (12)	96.4	91.9 (12)
	AWisc/2005	n.a.	n.a.	80.9 (15)	91.9	86.6 (15)	86.3 (18)	95.0	91.7 (10)
	A/Perth/2009	n.a.	n.a.	80.5 (17)	91.4	86.0 (14)	87.6 (14)	94.1	91.3 (11)
	A/Vict/2011	n.a.	n.a.	80.5 (16)	91.4	84.7 (17)	86.1 (15)	94.1	91.3 (8)

*HA* hemagglutinin, *NA* neuraminidase, *Eu* European, *N A* North American, hu = human seasonal H3N2 influenza virus, *n.a*. not applicable

*Number of aa differences at presumed antigenic sites is indicated between brackets. Full virus names: sw/Gent/1/1984, sw/England/163266/1987, sw/Gent/172/2008, sw/Gent/26/2012, sw/Texas/4199-2/1998, sw/Minnesota/593/1999, sw/Ontario/33853/2005, sw/Pennsylvania/A01076777/2010, A/Indiana/08/2011, sw/Iowa/A01049750/2011, A/Victoria/3/1975, A/England/427/1988, A/Nanchang/933/1995, A/Wisconsin/67/2005, A/Perth/16/2009, A/Victoria/361/2011

**Table 2:** Comparison of HI and VN antibody titers against heterologous H3N2 influenza viruses in pigs of different vaccine groups

	

1°, 2°: Virus strains used for first and second vaccination; Antibody titers were determined 2 weeks after the last vaccination (day 42); See table 1 for full virus names, *cluster I or IV HA indicated between brackets; *n* = number of pigs examined per group.

Sera were tested at an initial dilution of 1:20 (HI) or 1:16 (VN), those that were negative were assigned a titer of 10 (HI) or 8 (VN); Grey shade indicates titers ≥ 40 in HI assay and ≥64 in VN assay, which are considered as seroprotective.

The color coding of viruses is based on the antigenic clusters of HA.^2, 4, 26^ Viruses of different clusters differ by at least 4 antigenic units.

The genetic relatedness between the surface proteins of the vaccine and test viruses was in line with the ancestry of the vaccine strains (Table 1). According to previous studies with human and swine H3N2 viruses marked antigenic differences and vaccine failure may result from as few as 1 or 2 substitutions at 6–7 key aa residues in antigenic sites A and B.^4, 27^ Most viruses included in HI and VN assays differed from both vaccine strains in at least 4 of these residues (Figs. S1, 2). While the HA2 was more conserved, the NA showed a largely similar pattern as the HA1. However, the NA of G08 and PA10 showed considerable genetic divergence from that of sw/Gent/26/2012 (H1N2) and sw/Iowa/A01049750/2011 (H3N2) respectively.

Mock-vaccinated control pigs tested negative against all viruses in all three assays. The vaccinated pigs showed largely similar antibody profiles in HI and VN assays, though VN titers were usually higher (Table 2). Based on the published literature ^28, 29^ we consider HI titers ≥ 40 and VN titers ≥ 64 as seroprotective. The homologous prime-boost vaccinations with G08 or PA10 induced seroprotective antibody titers against SIVs of the same lineage as the vaccine strains and related human seasonal H3N2 viruses, but not to human viruses from 2005–2011. The heterologous prime-boost group, on the other hand, developed antibodies against both European and North American cluster IV H3N2 SIVs, though antibody titers were generally lower than in the respective homologous prime-boost groups. Only this group mounted solid antibody titers against the more divergent cluster I North American H3N2 SIVs sw/Texas/4199-2/1998 and sw/Minnesota/593/1999. Remarkably, two administrations of bivalent vaccine induced barely detectable antibody titers against cluster I viruses and lower titers against some human H3N2 viruses than the heterologous prime-boost. As expected, a single administration of bivalent vaccine was insufficient to induce seroprotective antibody titers.

NI assays were performed against a smaller selection of viruses (Table 3). The homologous prime-boost vaccinations with G08 or PA10 induced high NI titers against the respective vaccine strains and against the related IN11 strain in the PA10 homologous prime-boost group. Antibody titers against SIVs with an antigenically distinct NA were ≥10-fold lower or undetectable. After the heterologous prime-boost vaccination, in contrast, most pigs had substantial NI titers against both vaccine strains and the four heterologous SIVs. Most remarkable were the high NI titers (GMT 160) against the European H1N2 SIV sw/Gent/26/2012, which exceeded those in any other group including the bivalent vaccine (2x) group.

**Table 3:** Comparison of NI antibody titers against heterologous H3N2 and H1N2 influenza viruses in pigs of different vaccine groups

		Geometric mean antibody titer (no. of pigs with detectable titers)				
	1°	G08	PA10	G08	–	G08+PA10
	2°	G08	PA10	PA10	G08+PA10	G08+PA10
Virus lineage/strain		*n* = 6	*n* = 6	*n* = 6	*n* = 6	*n* = 4
*Eu swine N2*						
Sw/Gent/2008 (H3N2)		1140 (6)	36 (4)	806 (6)	10	320 (4)
Sw/Gent/2012 (H1N2)		22 (3)	50 (5)	160 (6)	10	95 (4)
*N A swine H3N2*						
Sw/TX/1998 (hu ’95-’96)*		11 (1)	25 (3)	57 (5)	10	34 (4)
Sw/PA/2010 (hu ’01-’02)		11 (1)	806 (6)	143 (6)	10	381 (4)
A/IN/2011 (hu ’01-’02)		10	453 (6)	57 (5)	10	320 (4)
Sw/IA/2011 (hu ’95-’96)		20 (4)	80 (6)	45 (6)	10	67 (4)
*Human H3N2*						
A/Perth/2009		10	11 (1)	13 (1)	10	10
A/Victoria/2011		10	10	11 (1)	10	10

1°, 2°: Virus strains used for first and second vaccination; Antibody titers were determined 2 weeks after the last vaccination (day 42); See table 1 for full virus names, *origin of the NA indicated between brackets, hu = human seasonal H3N2 influenza virus; *n* = number of pigs examined per group

Sera were tested at an initial dilution of 1:20, those that were negative were assigned a titer of 10

Overall, the sera from the heterologous prime-boost group were consistently the most cross-reactive in HI, VN, and NI assays (Fig. S4).

### Lack of HA-stalk and heterosubtypic antibodies after heterologous prime-boost vaccination

The finding that VN antibody titers were several fold higher than HI titers suggests that antibodies may be in part directed to regions outside the receptor-binding site of the HA. We therefore examined postvaccination sera for antibody specific for the HA stalk by ELISA. However, while antistalk titers were measurable in vaccinated pigs they ranged from 100 to 400 in all groups without statistically significant differences with the mock-vaccinated control group (Fig. S5, *P* > 0.05). These titers are very low and unlikely to mediate protection. As comparison, adult humans have baseline titers of 1600 which are considered non-protective.^30, 31^

Finally, we performed HI and VN assays against avian H4 and H7 influenza viruses, which are phylogenetically closely related to H3, as well as H1 SIVs. Heterosubtypic antibodies were detectable in the heterologous prime-boost group only (Table S3). Low antibody titers against H1N2 were found in two pigs by HI and in four pigs by VN. Only one pig showed antibodies against H4N1 and H4N6 viruses. All pigs of this group were negative for antibodies against H1N1 and H7N1 viruses.

The overall lack of heterosubtypic antibodies is consistent with the lack of antistalk antibodies and suggests that antibodies induced by the heterologous prime-boost vaccination primarily target conserved epitopes in the globular head of the HA.

## Discussion

We have proven the concept that priming and boosting pigs with inactivated SIVs of distinct H3N2 lineages can broaden the immune response against both HA and NA. Broader anti-HA antibody responses and protection have previously been reported after sequential immunizations of humans, birds, ferrets or mice with antigenically distinct viruses within the H1N1, H3N2 or H5 subtype.^15–20^ Previous studies in pigs have shown enhanced anti-HA antibody titers after priming with live wild-type SIVs, or HA gene-based vaccines, and boosting with a single dose of commercial inactivated SIV vaccine.^32–34^ But while the HAs of the viruses used for both immunizations were divergent in the studies with live virus,^34^ they were very similar in those with gene-based vaccines.^32, 33^ The present study is also the first to examine antibody responses to both HA and NA of swine and human H3N2 influenza viruses from various clusters. The observed anti-N2 antibody response may even partially protect against the European H1N2 SIV lineage, for which both surface proteins are far distant from the co-circulating H1N1 and H3N2 SIVs. On the other hand, our approach failed to induce antibodies against the most recent human seasonal H3N2 viruses or other subtypes, and it does not alleviate the need for H1 components in SIV vaccines. This is in agreement with the minimal anti-H3 stalk antibody titers, which appear to be essential for cross-reactivity across subtypes and cannot be induced by whole inactivated vaccines as such.^35^

Based on the published literature,^15, 21, 36, 37^ we speculate that sequential vaccination with G08 and PA10 induces antibodies against shared epitopes in the HA of both viruses. The traditional approach of using the same or very similar influenza strains for the first and secondary vaccinations is known to favor an isolate-specific antibody response against highly variable, immunodominant epitopes in the globular head of the HA. Antigenically very distinct strains within the same subtype, such as G08 and PA10, differ from each other in most or many of these variable epitopes, which are recognized by the majority of memory B cells induced by the previous vaccination(s). It is believed that a booster vaccination with such a distinct strain has the capacity to expand the rare memory B cells that recognize conserved, subdominant epitopes in the HA head or stalk.^15, 21, 36, 37^ Consequently, the resulting antibodies will not only cross-react with both vaccine strains, they will likely also be more cross-reactive against other isolates. The overall broader serologic cross-reactivity after heterologous prime-boosting with G08 and PA10 than after two administrations of bivalent vaccine (Fig. S4) supports this hypothesis. Our ELISPOT analyses of influenza-specific ASC show limitations regarding the number of pigs, timepoints and viruses examined. Also, the respective homologous prime-boost groups showed higher responses to PA10 than to G08 after the booster vaccination. We should mention here that we had unwillingly used a slightly lower concentration of G08 than of both other test viruses, which may have resulted in an underestimation of the response to G08. Still, the heterologous boost led to an increase in ASC against all three viruses examined. We cannot explain why two pigs of the heterologous prime-boost group had higher ASC responses than the homologous prime-boost controls without showing higher serum antibody titers. It is possible that the kinetics of antibody secretion differ after heterologous vs. homologous boosting and/or that the ELISPOT detects a broader spectrum of antibodies than the functional serological assays. Our findings are reminiscent of the rapid and broad immune response to a single dose of subunit 2009 pandemic H1N1 influenza vaccine in people without detectable antibodies to this antigenically novel virus.^21^ Their ASC responses peaked as early as 1 week post vaccination and consisted mainly of IgG secreting plasmablasts that had undergone extensive affinity maturation.^21, 37^ This led to the discovery of pre-existing cross-reactive memory B cells that bind to epitopes conserved between past seasonal H1N1 strains from 1977–2008 and the pandemic H1N1 virus, and which appear to be most efficiently recalled by consecutive immunizations with divergent virus strains. ^21, 37, 38^

The heterologous prime-boost vaccination offered a superior protection against challenge with G08 as compared to PA10, which was associated with higher anti-G08 serum antibody titers. This raises the question as to whether the heterologous boost favours an antibody response to the virus strain encountered first. A similar phenomenon exists in humans and laboratory animals and has been called “back boosting”, “antigenic seniority” or, initially, “original antigenic sin”.^39–42^ Consistent with other studies with inactivated influenza vaccine,^43^ our findings do not fit the strictest definition of original antigenic sin, because there was no impairment or true suppression of the antibody response to the second vaccine strain. Also, the difference in antibody titers against both vaccine viruses was most pronounced in VN and NI assays, and minimal in the HI assay. Previous studies with influenza as well as HIV indicate that the prime-boost response may be affected by the order and timing of immunizations and by the vaccine formulations.^16–18, 20, 38, 44, 45^ Yet the key question remains whether the concept applies to any combination of sufficiently distant H3N2 viruses. Affirmative results come from studies using different combinations of inactivated, adjuvanted H1N1 or H5 influenza viruses, and different immunization schemes, in humans, zoo birds and laboratory animals.^17, 18, 20, 38, 46, 47^ These studies mostly used subunit vaccines and the human-approved oil-based adjuvants MF59 and AS03, which appear to be critical for the efficacy of the prime-boost regimes. Both adjuvants have been shown to drive the expansion of memory B cells that can recognize variant influenza strains not included in the original vaccine.^17, 38, 46^ The Emulsigen® adjuvant used in our study is also an oil-in-water emulsion and will presumably have similar effects.

Other researchers have reported enhanced lung lesions after vaccination of pigs with whole inactivated SIV and challenge with heterologous strains of the same subtype.^25^ This so-called “vaccine-associated enhanced respiratory disease” is characterized by increased gross lung lesions and microscopic lung and tracheal lesion scores in the vaccinated pigs as compared to unvaccinated challenge controls. It has been attributed to non-neutralizing virus-binding antibodies and has raised concern about the use of inactivated SIV vaccines and the design of universal influenza vaccines for humans.^48^ The studies describing VAERD generally used the more aggressive intratracheal inoculation route, whereas we used intranasal inoculation. In any case it is reassuring that none of the vaccine groups in our study showed enhanced respiratory tract pathology.

In summary, we have demonstrated that priming and booster vaccinations with antigenically very distinct, geographically separated H3N2 SIV lineages can induce protection against both and increase cross-cluster serologic reactivity. It remains to be examined whether our findings extend to other combinations of antigenically divergent H3N2 viruses, or what is the optimal genetic distance between viruses, and whether it is possible to induce a pan-H3N2 protection against past and future strains of both swine and humans. Ideally, the heterologous H3N2 boost would eliminate the need for two doses of bivalent H3N2 vaccine and thus reduce the total amount of traditional SIV vaccine needed by half and production costs by 25–30%.

## Materials and methods

### Vaccine preparation

G08 is representative of the European H3N2 SIV lineage, it was isolated from an outbreak of acute respiratory disease in fattening pigs in East Flanders, Belgium. PA10 is a cluster IV North American H3N2 SIV and was obtained from the US Department of Agriculture (USDA) swine influenza repository held at the National Veterinary Service Laboratories.

Virus stocks were generated by inoculation of Madin-Darby Canine Kidney (MDCK, ATCC CCL-34) cells with 1 ml of virus (approx. 10^6.3^ TCID_50_ per ml, first passage in MDCK cells) in a volume of 10 ml minimal essential medium containing supplements (2 mM L-glutamine, 1 mg/ml lactalbumin, 100 U/ml penicillin, 100 μg/ml streptomycin, 50 μg/ml gentamycin and 2 μg/ml trypsin) in T75 flasks for 2 h at 37 °C, 5% CO2. Subsequently, fresh medium was added and the cells were further incubated for at least 24 h or until 40–50% cytopathogenic effect was observed. After one freeze-thaw cycle, medium containing virus was clarified by centrifugation (2 × 10 min, 1831 g) and stored at −70 °C in 10 ml aliquots. Vaccine virus stocks had a titer of 10^8.3^ TCID_50_ per ml. MDCK cells were free of Mycoplasma contamination in a PCR detection kit (Sigma-Aldrich, St. Louis, MO, USA, MP0035).

For ultraviolet light (UV) inactivation, 10 ml aliquots of virus were thawed, placed in 60 mm Petri dishes to a fluid depth of 5 mm and exposed to 120 J/cm^2^ from a UV source (Ultra-Violet Products Ltd., Cambridge, UK) for 10 min. The dishes were placed on ice at a distance of 10 cm from the light source. Loss of viral infectivity was confirmed by two serial passages in MDCK cells. One milliliter aliquots of inactivated virus were stored at −70 °C for later analysis in a standard HA assay with turkey erythrocytes.^49^ HA titers were 2560 hemagglutinating units (HAU) per ml before and after inactivation. Each 2 ml vaccine dose contained 256 HAU of inactivated G08 and/or PA10 virus diluted in phosphate-buffered saline (PBS) and 20% commercial oil-in-water adjuvant (Emulsigen®, MVP Laboratories, NE, USA). The inactivation method, adjuvant and antigen dose had previously been shown to be appropriate for immunization of pigs against SIVs.^4, 25, 48^

### Experimental design and evaluation of protection against challenge

Sixty-five 3-week-old conventional pigs which were free of influenza A virus antibodies were divided into six groups (Table S2) and vaccinated intramuscularly at 4 and/or 8 weeks of age. A mock-vaccinated challenge control group (*n* = 12) received two administrations of PBS with adjuvant. Two homologous prime-boost groups received two administrations of G08 (*n* = 12) and PA10 (*n* = 11) respectively. The heterologous prime-boost group (*n* = 14) was primed with G08 and boosted with heterologous PA10 vaccine. Two bivalent vaccine groups were vaccinated with a combination vaccine containing both G08 and PA10 at the age of 8 weeks only (*n* = 12) or at 4 and 8 weeks (*n* = 4). Four additional pigs served as unvaccinated unchallenged controls to assess histological characteristics of the respiratory tract of healthy pigs of the same cohort. Animals were randomly assigned to experimental groups. Sample size was calculated to allow a significance of *P* < 0.05 and 80% power.

One month after the booster vaccination all pigs, except unvaccinated unchallenged controls, were challenged intranasally with 10^7^ TCID_50_ SIV. Half of the pigs in each group were challenged with G08 and half with PA10. Each vaccination-challenge group was housed in a separate BSL2 + isolation room. One pig in the G08 homologous prime-boost group died from reasons unrelated to the experiment in the week before challenge, leaving 11 pigs for challenge in this group. Three days post challenge all pigs were euthanized to evaluate virus titers in 20% tissue homogenates of the nasal mucosa, trachea and three different lung samples representing the entire lung, as well as (histo)pathological lesion scores of the trachea and lungs.

Virus titrations were performed in MDCK cells.^50^ Lesions of the lung and trachea were analyzed and scored as described by Vincent et al.^25^ Laboratory analyses were performed blinded. All experiments were performed at the Faculty of Veterinary Medicine, Ghent University, and approved and supervised by its Ethical Committee.

### Serum antibody responses

Serum samples were collected immediately before the priming and booster vaccinations and 2 and 4 weeks after the booster (weeks 0, 4, 6, 8) to determine HI, VN, and NI antibody titers against G08 and PA10. All sera were examined in HI and VN assays, and 4 to 8 sera per group in NI assays.

Two weeks after the last vaccination 4 to 6 sera per group were analysed for HI, VN, and NI titers against swine and human influenza viruses with an antigenically distinct H3 or N2 (Tables 1–3). HA1, HA2, and NA sequences of these viruses were downloaded from GenBank and % aa identity with the sequences of the vaccine strains was determined with the MegAlign program and DNASTAR 5.01 software. Amino acid differences at putative antigenic sites of the HA1 and NA, as defined by others,^11, 22–24^ were identified by alignment using MEGA 6.06 software. The same sera were examined in an ELISA for antibodies against the H3 HA stalk of A/Perth/16/2009. Sera from the mock-vaccinated control, heterologous prime-boost and both homologous prime-boost groups were tested for HI and VN titers against European avian-like H1N1 (sw/Gent/28/2010) and human-like H1N2 (sw/Gent/26/2012) SIVs, and low pathogenic H4N1 (mallard/Alberta/47/1998), H4N6 (duck/Belgium/06936/2005) and H7N1 (chicken/Italy/1067/1999) avian influenza viruses.

HI, VN, and NI assays were performed according to standard procedures.^49–51^ For A/Perth/16/2009 and A/Victoria/361/2011, however, we used guinea pig instead of turkey red blood cells in the HI assay^52^ and MDCK-SIAT1 cells in the VN assay.^53^ Antibody titers were expressed as the reciprocal of the highest serum dilution that inhibited hemagglutination or virus replication, or that gave a 50% inhibition of NA activity. Sera were tested separately and in duplicate, at an initial dilution of 1:10 or 1:20 in HI and NI, and 1:2 or 1:16 in VN assays.

ELISAs were performed as described before^31^ with a secondary peroxidase-labeled rabbit anti-pig IgG antibody (Sigma-Aldrich, A5670) instead of anti-human IgG and a serum starting dilution of 1:100. Two substrates were used: recombinant cH5/3 HA protein with the stalk domain of A/Perth/16/2009 (H3N2) and the head domain of A/Vietnam/1203/2004 (H5N1) and trimeric recombinant head-only protein from A/Vietnam/1203/2004, both produced in insect cells.^54, 55^ Optical density values measured with the head-only construct were subtracted from the values measured with the cH5/3 protein. Endpoint titers were calculated using the average of blank wells plus 3 standard deviations as cut off.

### ELISPOT analysis of antibody secreting cells

Heparinized whole blood for isolation of PBMC^56^ was collected at the time of the last vaccination and 7 days later from four pigs per group, except for the bivalent vaccine (2x) group. An ELISPOT assay to determine IgG ASC specific for G08, PA10 and IN11 was performed as described by Kitikoon et al.^56^ Briefly, 96-well ELISPOT filter plates (MAIPS4510, Millipore Corp., MA, USA) were coated overnight with 200 HAU per well of live purified influenza virus. Freshly isolated PBMC (5 × 10^5^ cells per well) were dispensed into duplicate wells. After 18 h incubation (37 °C, 5% CO_2_) mouse anti-swine IgG monoclonal antibody (clone 27.8.1, CVI Lelystad, The Netherlands) was added followed by HRP-labeled goat anti-mouse immunoglobulins (Dako, Glostrup, Denmark, P0447). ASC were visualized using the TMB Liquid Substrate System for Membranes (Sigma-Aldrich) and counted with an ELISPOT plate reader. Non-specific spots detected in wells coated with mock-infected MDCK cell medium were subtracted from the counts of influenza-specific ASC.

### Statistical analysis

Antibody titers against the vaccine strains, numbers of ASC and lesion scores were compared between groups using the Kruskal-Wallis test. Analysis of variance (ANOVA) was used to compare virus titers, and Fisher’s exact test for virus isolation rates and numbers of pigs with lesions. Kruskal-Wallis was followed by Dunn’s test, and ANOVA by Dunnett’s test. Samples that tested negative in serological assays were assigned a value corresponding with half of the minimum detectable antibody titer. Samples that tested negative for virus were given a value of 0.85 log_10_ TCID_50_. Spearman’s rank correlation coefficient was used to assess the relationship between virus titers and lesion scores.

## Electronic supplementary material

Fig S1

Fig S2

Fig S3

Fig S4

Fig S5

Table S1

Table S2

Table S3

Supplementary Figure Legends
