# Supplementary material for: Heterologous prime-boost vaccination with H3N2 influenza viruses of swine favors cross-clade antibody responses and protection
Source: NPJ Vaccines. 2017 Apr 20;2:11. doi: 10.1038/s41541-017-0012-x (PMC5604745; doi:10.1038/s41541-017-0012-x)
Supplement: Supplementary file 1 — Fig S1 [file 41541_2017_12_MOESM1_ESM.pdf]

```
                                                                                      C         E         70

A/swine/Gent/172/2008         QDLPGKGNNT ATLCLGHHAV PNGTLVKTIT DDQIEVTSAT ELVQNFSMGK ICKNPHRILD GANCTLIDSL
A/swine/Gent/1/1984           ....EN..S. .K........ .......... N......N.. ....S..... ..N....V.. ........A.
A/swine/England/163266/1987   .GFSRNDD.I .......... .......... N......N.. ....SS.T.. ..N....... .MD...V.A.
A/swine/Texas/4199-2/1998     .K...ND.S. .......... .......... N......N.. ....SS.T.R ..DS...... .K......A.
A/swine/Minnesota/593/1999    .K...ND.S. .......... .......... N......N.. ....SS.T.R ..DS...... .K......A.
A/swine/Ontario/33853/2005    .K...SD.SM .......... .......... .......N.. ....SS.T.R ..NS..Q... .K......A.
A/swine/Penn/A01076777/2010   .K...SD.S. .......... .......... .......N.. ....SS.T.R ..NS..Q... .K......A.
A/Indiana/08/2011             .K...SD.SM .......... .......... .......N.. ....SS.T.G ..NS..Q... .K......A.
A/swine/Iowa/A01049750/2011   .K...ND.SM .......... .......... ...V...N.. ....SS.T.R ..NS..Q... .K......A.
A/Victoria/3/1975             .....ND.S. .......... .......... N......N.. ....SS.T.. ..N....... .I......A.
A/England/427/1988            .K...ND.S. .......... .......... N......N.. ....SS.T.R ..DS...... .K......A.
A/Nanchang/933/1995           .K...ND.S. .......... .......... N......N.. ....SS.T.R ..DS...... .K......A.
A/Wisconsin/67/2005           .K...ND.S. .......... ....I..... N......N.. ....SS.T.G ..DS..Q... .E......A.
A/Perth/16/2009               .K...ND.S. .......... ....I..... N......N.. ....SS.T.E ..DS..Q... .K......A.
A/Victoria/361/2011           .K...ND.S. .......... ....I..... N......N.. .....S.I.E ..DS..Q... .E......A.

                                     E   E E                                                      A   A  A140

A/swine/Gent/172/2008         LGDPHCDGFQ NEKWDLFIER SKAFSNCYPY DVPEYTSLRSL IASSGTLEFT NEDFNWTGV TQNGGSSACK
A/swine/Gent/1/1984           .......... .......V.. .......... ...D.A..... .........I ..G...... ......N...
A/swine/England/163266/1987   .......... ..T....V.. .......... ...D.A..... V........I ..G...V.. ..........
A/swine/Texas/4199-2/1998     .......... .KE....... ...Y...... ...D.S..... V......... ......... A.D...YS..
A/swine/Minnesota/593/1999    .......... .KE....... ...Y...... ...D.S..... V......... ......... A.D...YS..
A/swine/Ontario/33853/2005    ........D. .KE....V.. .T.Y...... Y..D.A..... V......... Q.S...... A.D.S.Y..R
A/swine/Penn/A01076777/2010   ........D. .KE....V.. .T.Y...... Y..D.V..... V......... Q.N...... A.D.S.Y..R
A/Indiana/08/2011             ........D. .KE....V.. .T.Y...... Y..D.AT.... V....N.... Q.S...... A.G.S.Y..R
A/swine/Iowa/A01049750/2011   ........E. .TE....V.. .T.Y...... Y..D.S..... V......... Q.S.D.... A.D.S.Y...
A/Victoria/3/1975             .......... .......V.. .......... ...D.A..... V........I ..G...... ......N...
A/England/427/1988            .......... .KE....V.. ..Y....... ...D.A..... V........I ......... A.S...Y...
A/Nanchang/933/1995           .......... .KE....V.. ..Y....... ...D.A..... V......... ..G...... A.D.T.Y...
A/Wisconsin/67/2005           .....Q.... .K.....V.. ..Y....... ...D.A..... V........N D.S...... .....T..S.
A/Perth/16/2009               .....Q.... .K.....V.. ..Y....... ...D.A..... V........N ..S...... .....T...I
A/Victoria/361/2011           .....Q.... .K.....V.. ..Y....... ...D.A..... V........N ..S...... .....T...I

                                A               B                               B      B        D   D D    210

A/swine/Gent/172/2008         RGPNNSFFSR LNWLYKSGNT YPMLNVTMPN SDDFDKLYIW GVHHPSTDRE QTNLYIQASG KITVSTKRSQ
A/swine/Gent/1/1984           ....S..... .......... .......... ..N....... .......... .....V.... .V..F....H
A/swine/England/163266/1987   ..SDS..... .......... ..V....... N.N....... .I.......Q. ..R..ALE.. RV........
A/swine/Texas/4199-2/1998     ..SVK..... ....H.LEYK ..A....... N.K....... ........S. ..S..V..I. RV......S..
A/swine/Minnesota/593/1999    .ESVK..... ....H.LEYK ..A....... N.K....... ........S. ..S..V..I. RV......S..
A/swine/Ontario/33853/2005    .ESV...... ....HNLDYK ..A....... N.K....... .....G...D .....V.... RV........
A/swine/Penn/A01076777/2010   .KSV...... .....NLNYK ..A....... N.N....... .....G..KD .......... RV........
A/Indiana/08/2011             ..SV...... .....NLNYK ..EQ...... N.K....... .....G..KD .....V.... RVI.......
A/swine/Iowa/A01049750/2011   ..SV...... ....HNLNYK ..A....... N.N....... .....G...D .A...V.... RV........
A/Victoria/3/1975             ...DIG.... .........S. ..VQ...... N.NS...... ........K. ..D..V.... .V........
A/England/427/1988            ..SV...... ....H..EYK ..A....... NGK....... .......... .....VR... RV........
A/Nanchang/933/1995           ..SVK..... ....H.LEYK ..A....... N.K....... ........SD ..S..V.... RV........
A/Wisconsin/67/2005           .RS....... ....THLKFK ..A....... NEK....... .....V..ND .IF..A.... R.........
A/Perth/16/2009               .RSK...... ....THLNFK ..A....... NEQ....... ..L..G..KD .IF..A.... R.........
A/Victoria/361/2011           .RS....... ....THLNFK ..A....... NEQ....... .....G..KD .IF..A.S.. R.........

                                    D  D                                                               C   280

A/swine/Gent/172/2008         QTIIPNIGSR PWVRGLSSRI SIYWTIVKPG DILIINSNGN LIAPRGYFKI QTGKSSVMRS DAPIGTCNSE
A/swine/Gent/1/1984           ......V..G .......... .......... ...V...... .........M HN.R..I... ........S..
A/swine/England/163266/1987   ..V...V.P. .......... .......... ...L...... .........M RA....II.. ....S..I..
A/swine/Texas/4199-2/1998     ..V....... .....I.... .......... ...L.S.T.. .......... RN....I... ....DN.Y..
A/swine/Minnesota/593/1999    ..V....... .....I.... .......... ...L.S.T.. .......... RN....I... ....DN.Y..
A/swine/Ontario/33853/2005    ..V....... .....V..I. .......... ...L...T.. .......... .S....I... .....N....
A/swine/Penn/A01076777/2010   ..V....... .....V..I. .......... ...L...T.. .......... .S....I... .....N.K..
A/Indiana/08/2011             ..V....... .....V..I. .......... ...L...T.. .......... .S....I... ..H.DE....
A/swine/Iowa/A01049750/2011   ..V....... .....V..V. .......... ...L...T.. .......... RN....I... ..H.DN....
A/Victoria/3/1975             ......V... .......... .......... ...V...... .........M R.....I... .......S..
A/England/427/1988            ..V....... .......... .......... ...L...T.. .......... R.....I... .......S..
A/Nanchang/933/1995           ..V....... .....I.... .......... ...L.K.T.. .......... RS....I... .....N....
A/Wisconsin/67/2005           ..V....... .RI.NIP... .......... ...L...T.. .......... RS....I... .....K....
A/Perth/16/2009               ..VS...... .R..NIP... .......... ...L...T.. .......... RS....I... .....K....
A/Victoria/361/2011           .AV....... .RI.NIP... .......... ...L...T.. .......... RS....I... .....K....
                                                                                    329

A/swine/Gent/172/2008         CITPNGSIPN DKPFQNVNRI TYGACPHYIK QNTLKLATGM RNIPERQTR
A/swine/Gent/1/1984           .......... ........K. ......K.V. .......... .....K...
A/swine/England/163266/1987   .......V.. ........K. ......K..R .......... .....K...
A/swine/Texas/4199-2/1998     .......... .......... ......K.V. .K........ ..V..K...
A/swine/Minnesota/593/1999    .......... .......... ......K.V. .K........ ..V..K...
A/swine/Ontario/33853/2005    .......... .......... ......R.V. .......... ..V..K...
A/swine/Penn/A01076777/2010   .......... .......... ...V..R.V. .......... ..V..K...
A/Indiana/08/2011             .......... ........K. ......R.V. .......... ..V..K...
A/swine/Iowa/A01049750/2011   .......... .......... ......R.V. .......... ..V..K...
A/Victoria/3/1975             .......... ........K. ......K.V. .......... ..V..K...
A/England/427/1988            .......... .......... ......R.V. .......... ..V..K...
A/Nanchang/933/1995           .......... .......... ......R.V. .......... ..V..K...
A/Wisconsin/67/2005           .......... .......... ......R.V. .......... ..V..K...
A/Perth/16/2009               .......... .......... ......R.V. .......... ..V..K...
A/Victoria/361/2011           .......... .......... ......R.V. .S........ ..V..K...
```
