# Supplementary material for: Heterologous prime-boost vaccination with H3N2 influenza viruses of swine favors cross-clade antibody responses and protection
Source: NPJ Vaccines. 2017 Apr 20;2:11. doi: 10.1038/s41541-017-0012-x (PMC5604745; doi:10.1038/s41541-017-0012-x)
Supplement: Supplementary file 2 — Fig S2 [file 41541_2017_12_MOESM2_ESM.pdf]

```
                                                                                     C         E         70

A/swine/Penn/A01076777/2010  QKLPGSDNST ATLCLGHHAV PNGTLVKTIT DDQIEVTNAT ELVQSSSTGR ICNSPHQILD GKNCTLIDAL
A/swine/Gent/1/1984          .D..ENG... .K........ .......... N......... .....F.M.K ...N..RV.. .A........
A/swine/England/163266/1987  .GFSRN.DNI .......... .......... N......... .........K ...N..R... .MD...V...
A/swine/Gent/172/2008        .D...KG.N. .......... .......... .......S.. ....NF.M.K ..KN..R... .A......S.
A/swine/Texas/4199-2/1998    .....N.... .......... .......... N......... .......... ..D...R... ..........
A/swine/Minnesota/593/1999   .....N.... .......... .......... N......... .......... ..D...R... ..........
A/swine/Ontario/33853/2005   .........M .......... .......... .......... .......... .......... ..........
A/Indiana/08/2011            .........M .......... .......... .......... .........G .......... ..........
A/swine/Iowa/A01049750/2011  .....N...M .......... .......... ...V...... .......... .......... ..........
A/Victoria/3/1975            .D...N..S. .......... .......... N......... .........K ...N..R... .I........
A/England/427/1988           .....N.... .......... .......... N......... .......... ..D...R... ..........
A/Nanchang/933/1995          .....N.... .......... .......... N......... .......... ..D...R... ..........
A/Wisconsin/67/2005          .....N.... .......... ....I..... N......... .........G ..D....... .E........
A/Perth/16/2009              .....N.... .......... ....I..... N......... .........E ..D....... ..........
A/Victoria/361/2011          .....N.... .......... ....I..... N......... ....N..I.E ..D....... .E........

                                    E   E E                                                      A   A  A140

A/swine/Penn/A01076777/2010  LGDPHCDDFQ NKEWDLFVER STAYSNCYPY YVPDYVSLRS LVASSGTLEF TQENFNWTGV AQDGSSYACR
A/swine/Gent/1/1984          .......G.. .EK....... .K.F...... D....A.... .I........ IN.G...... T.N.G.N..K
A/swine/England/163266/1987  .......G.. .ET....... .K.F...... D....A.... .......... IN.G...V.. T.N.G.S..K
A/swine/Gent/172/2008        .......G.. .EK....I.. .K.F...... D..E.T.... .I........ .N.D...... T.N.G.S..K
A/swine/Texas/4199-2/1998    .......G.. .......I.. .K........ D....S.... .......... .N.D...... ....G..S.K
A/swine/Minnesota/593/1999   .......G.. .......I.. .K........ D....S.... .......... .N.D...... ....G..S.K
A/swine/Ontario/33853/2005   .......... .......... .......... .....A.... .......... ...S...... ..........
A/Indiana/08/2011            .......... .......... .......... ......AT.. .......N.. ....S..... ..G.......
A/swine/Iowa/A01049750/2011  .......E.. .T........ .......... .....S.... .......... ...S.D.... .........K
A/Victoria/3/1975            .......G.. .EK....... .K.F...... D....A.... .......... IN.G...... T.N.G.N..K
A/England/427/1988           .......G.. .......... .K........ D....A.... .......... IN.D...... ..S.G....K
A/Nanchang/933/1995          .......G.. .......... .K........ D....A.... .......... .N.G...... ....T....K
A/Wisconsin/67/2005          ....Q..G.. ..K....... .K........ D....A.... .......... ND.S...... T.N.T.SS.K
A/Perth/16/2009              ....Q..G.. ..K....... .K........ D....A.... .......... NN.S...... T.N.T.S..I
A/Victoria/361/2011          ....Q..G.. ..K........ K........ D....A.... .......... NN.S...... T.N.T.S..I

                               A             B                                B       B        D   D D  210

A/swine/Penn/A01076777/2010  RKSVNSFFSR LNWLYNLNYK YPALNVTMPN NDNFDKLYIW GVHHPGTDKD QTNLYIQASG RVTVSTKRSQ
A/swine/Gent/1/1984          .GPNS..... .....KSGNT ..M....... S......... .....S..RE .....V.... K...F....H
A/swine/England/163266/1987  .G.DS..... .....KSGNT ..V....... .......... .I...S..QE ..R..ALE.. ..........
A/swine/Gent/172/2008        .GPN...... .....KSGNT ..M....... S.D....... .....S..RE .......... KI........
A/swine/Texas/4199-2/1998    .G..K..... ....HK.E.. .......... ..K....... .....S..SE ..S..V..I. .......S..
A/swine/Minnesota/593/1999   .E..K..... ....HK.E.. .......... ..K....... .....S..SE ..S..V..I. .......S..
A/swine/Ontario/33853/2005   .E........ ....H..D.. .......... ..K....... ........R. .....V.... ..........
A/Indiana/08/2011            .G........ .......... ..EQ...... ..K....... .......... .....V.... ..I.......
A/swine/Iowa/A01049750/2011  .G........ ....H..... .......... .......... ........R. .A...V.... ..........
A/Victoria/3/1975            .GPDIG.... .....KSGST ..VQ...... ...S...... .....S...E ..D..V.... K.........
A/England/427/1988           .G........ ....HKSE.. .......... .GK....... .....S..RE .....VR... ..........
A/Nanchang/933/1995          .G..K..... ....HK.E.. .......... ..K....... .....S..S. ..S..V.... ..........
A/Wisconsin/67/2005          .R.N...... ....TH.KF. .......... .EK....... .....V..N. .IF..A.... .I........
A/Perth/16/2009              .R.K...... ....TH..F. .......... .EQ....... ..L....... .IF..A.... .I........
A/Victoria/361/2011          .R.N...... ....TH..F. .......... .EQ....... .......... .IF..A.S.. .I........

                                   D  D                                                                C   280

A/swine/Penn/A01076777/2010  QTVIPNIGSR PWVRGVSSII SIYWTIVKPG DILLINSTGN LIAPRGYFKI QSGKSSIMRS DAPIGNCKSE
A/swine/Gent/1/1984          ..I...V..G .....L..R. .......... ...V...N.. .........M HN.R...... .....T.S..
A/swine/England/163266/1987  ......V.P. .....L..R. .......... .......N.. .........M RA.....I.. ....ST.I..
A/swine/Gent/172/2008        ..I....... .....L..R. .......... ...I...N.. .......... .T....V... .....T.N..
A/swine/Texas/4199-2/1998    .......... .....I..R. .......... .....S.... .......... RN........ ....D..Y..
A/swine/Minnesota/593/1999   .......... .....I..R. .......... .....S.... .......... RN........ ....D..Y..
A/swine/Ontario/33853/2005   .......... .......... .......... .......... .......... .......... .......N..
A/Indiana/08/2011            .......... .......... .......... .......... .......... .......... ..H.DE.N..
A/swine/Iowa/A01049750/2011  .......... ........V. .......... .......... .......... RN........ ..H.D..N..
A/Victoria/3/1975            ..I...V... .....L..R. .......... ...V...N.. .........M RT........ .....T.S..
A/England/427/1988           .......... .....L..R. .......... .......... .......... RT........ .....T.S..
A/Nanchang/933/1995          .......... .....I..R. .......... .....K.... .......... R......... .......N..
A/Wisconsin/67/2005          .......... .RI.NIP.R. .......... .......... .......... R......... .....K.N..
A/Perth/16/2009              ...S...... .R..NIP.R. .......... .......... .......... R......... .....K.N..
A/Victoria/361/2011          .A........ .RI.NIP.R. .......... .......... .......... R......... .....K.N..

                                                                                   329

A/swine/Penn/A01076777/2010  CITPNGSIPN DKPFQNVNRI TYGVCPRYVK QNTLKLATGM RNVPEKQTR
A/swine/Gent/1/1984          .......... ........K. ...A..K... .......... ..I......
A/swine/England/163266/1987  .......V.. ........K. ...A..K.IR .......... ..I......
A/swine/Gent/172/2008        .......... .......... ...A..H.I. .......... ..I..R...
A/swine/Texas/4199-2/1998    .......... .......... ...A..K... .K........ .........
A/swine/Minnesota/593/1999   .......... .......... ...A..K... .K........ .........
A/swine/Ontario/33853/2005   .......... .......... ...A...... .......... .........
A/Indiana/08/2011            .......... ........K. ...A...... .......... .........
A/swine/Iowa/A01049750/2011  .......... .......... ...A...... .......... .........
A/Victoria/3/1975            .......... ........K. ...A..K... .......... .........
A/England/427/1988           .......... .......... ...A...... .......... .........
A/Nanchang/933/1995          .......... .......... ...A...... .......... .........
A/Wisconsin/67/2005          .......... .......... ...A...... .......... .........
A/Perth/16/2009              .......... .......... ...A...... .......... .........
A/Victoria/361/2011          .......... .......... ...A...... .S........ .........
```
