# Supplementary figures and images for: Heterologous prime-boost vaccination with H3N2 influenza viruses of swine favors cross-clade antibody responses and protection
Source: NPJ Vaccines. 2017 Apr 20;2:11. doi: 10.1038/s41541-017-0012-x (PMC5604745; doi:10.1038/s41541-017-0012-x)

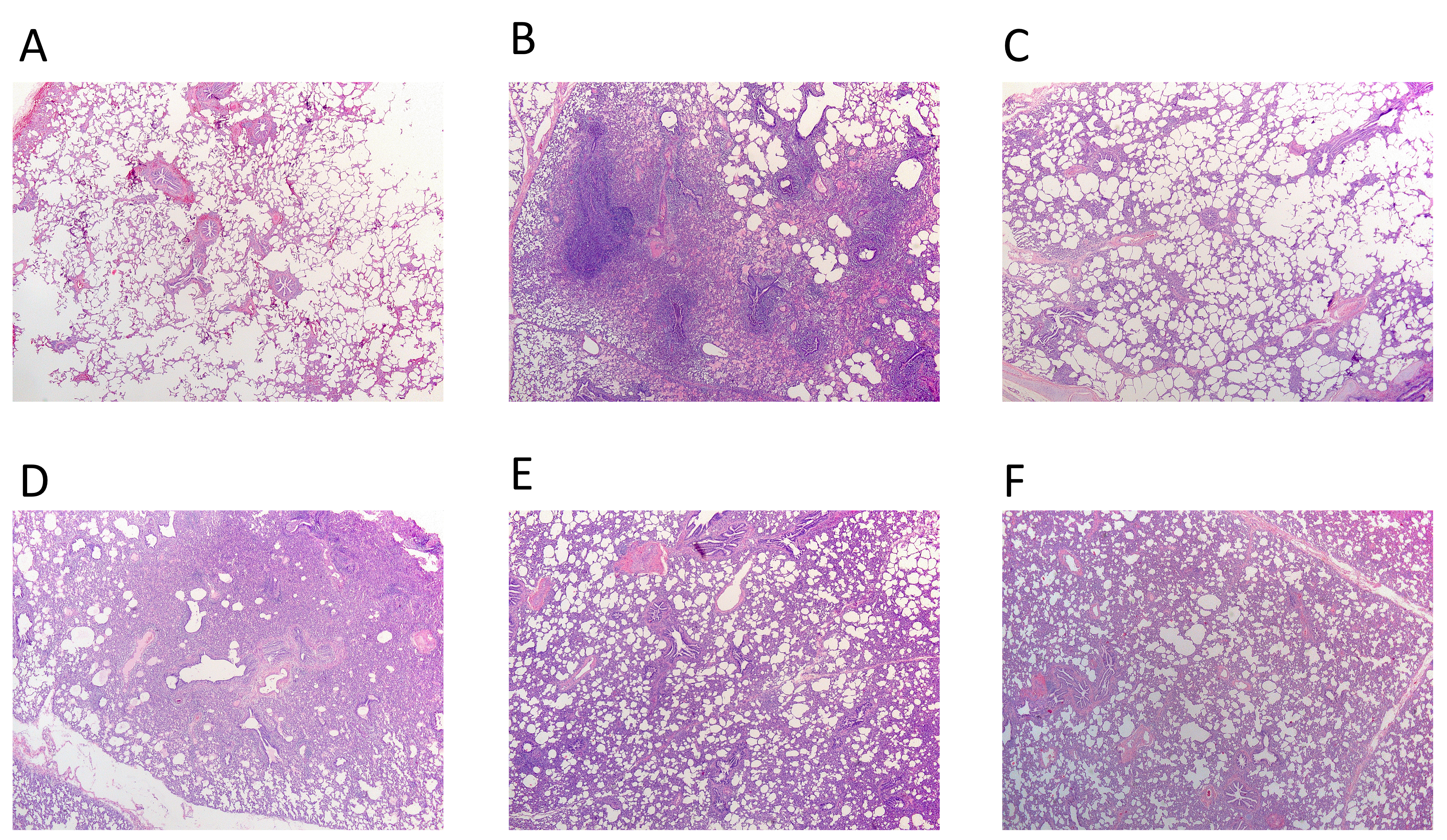

Supplement: Supplementary file 3 — Fig S3 [file 41541_2017_12_MOESM3_ESM.tif]

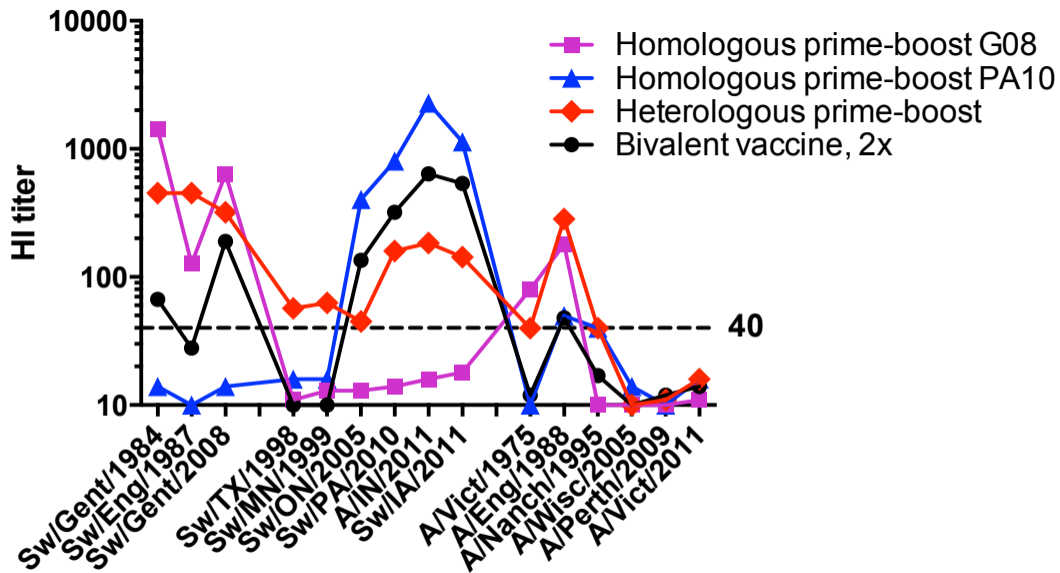

Eu swine H3N2

N A swine H3N2

Human H3N2

Supplement: Supplementary file 4 — Fig S4 [file 41541_2017_12_MOESM4_ESM.pdf]

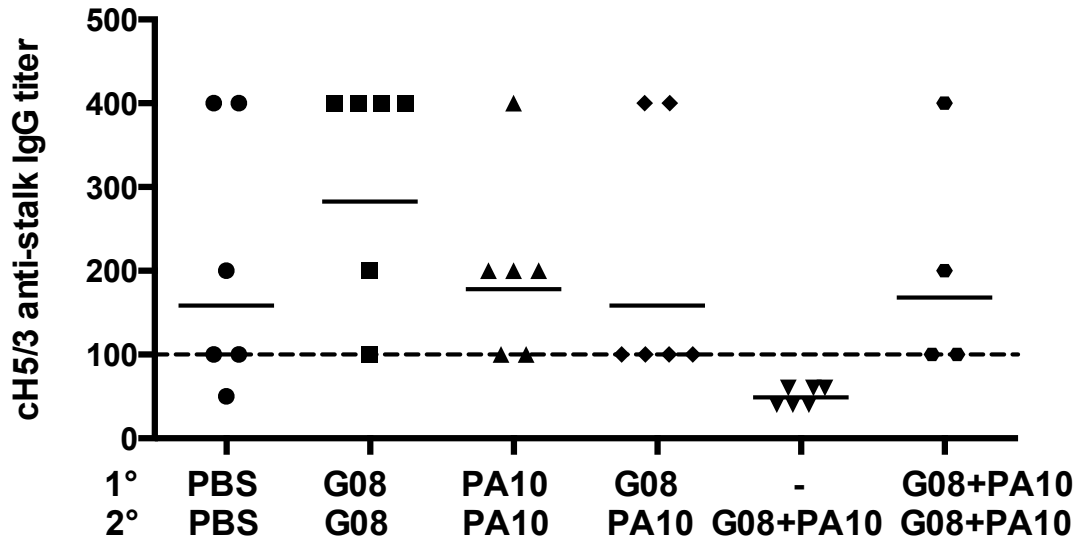

Supplement: Supplementary file 5 — Fig S5 [file 41541_2017_12_MOESM5_ESM.pdf]
