# Supplementary material for: Heterologous prime-boost vaccination with H3N2 influenza viruses of swine favors cross-clade antibody responses and protection
Source: NPJ Vaccines. 2017 Apr 20;2:11. doi: 10.1038/s41541-017-0012-x (PMC5604745; doi:10.1038/s41541-017-0012-x)
Supplement: Supplementary file 6 — Table S1 [file 41541_2017_12_MOESM6_ESM.docx]

**Table S1.** Comparison of serum HI, VN and NI antibody titers against the vaccine strains sw/Gent/172/2008 (G08) and sw/Pennsylvania/A01076777/2010 (PA10) in individual pigs of the heterologous prime-boost and bivalent vaccine (2x) group

|  |  |  | Antibody titer | | | | | | | | | | | | | | |
| --- | --- | --- | --- | --- | --- | --- | --- | --- | --- | --- | --- | --- | --- | --- | --- | --- | --- |
|  |  |  | Week 6 | | | | | | |  | Week 8 | | | | | | |
|  |  |  | G08 | | |  | PA10 | | |  | G08 | | |  | PA10 | | |
| Group | Pig nr. |  | HI | VN | NI |  | HI | VN | NI |  | HI | VN | NI |  | HI | VN | NI |
| Heterologous prime-boost G08-PA10 | 1884 |  | 160 | 3072 | 640 |  | 80 | 384 | 40 |  | 320 | 1024 | 320 |  | 160 | 512 | 160 |
|  | 1885 |  | 640 | 12288 | 1280 |  | 320 | 2048 | 320 |  | 640 | 4096 | 640 |  | 320 | 1536 | 320 |
|  | 1886 |  | 640 | 6144 | 2560 |  | 320 | 1536 | 640 |  | 640 | 4096 | n.t. |  | 640 | 768 | n.t. |
|  | 1887 |  | 160 | 3072 | 640 |  | 160 | 768 | 320 |  | 320 | 1024 | n.t. |  | 320 | 512 | n.t. |
|  | 1888 |  | 80 | 1536 | n.t. |  | 40 | 512 | n.t. |  | 80 | 512 | n.t. |  | 80 | 256 | n.t. |
|  | 1889 |  | 640 | 8192 | 2560 |  | 320 | 1024 | 320 |  | 640 | 6144 | 2560 |  | 1280 | 1536 | 320 |
|  | 1890 |  | 160 | 3072 | n.t. |  | 80 | 256 | n.t. |  | 160 | 768 | n.t. |  | 160 | 192 | n.t. |
|  | 1891 |  | 320 | 4096 | 1280 |  | 160 | 768 | 320 |  | 320 | 3072 | 160 |  | 160 | 384 | 40 |
|  | 1892 |  | 160 | 3072 | n.t. |  | 80 | 256 | n.t. |  | 160 | 512 | n.t. |  | 80 | 192 | n.t. |
|  | 1893 |  | 160 | 1536 | 320 |  | 80 | 384 | 160 |  | 160 | 768 | 320 |  | 160 | 512 | 320 |
|  | 1894 |  | 80 | 1536 | n.t. |  | 40 | 64 | n.t. |  | 80 | 192 | n.t. |  | 20 | 24 | n.t. |
|  | 1895 |  | 20 | 512 | n.t. |  | 20 | 32 | n.t. |  | 40 | 96 | n.t. |  | 40 | 64 | n.t. |
|  | 1896 |  | 320 | 4096 | 320 |  | 160 | 512 | 40 |  | 320 | 1536 | 320 |  | 160 | 512 | 320 |
|  | 1897 |  | 320 | 4096 | n.t. |  | 160 | 384 | n.t. |  | 320 | 1536 | n.t. |  | 160 | 384 | n.t. |
|  | **Mean** |  | **195** | **3083** | **905** |  | **108** | **410** | **190** |  | **226** | **1059** | **453** |  | **160** | **339** | **202** |
| Bivalent vaccine G08+PA10 (2x) | 2073 |  | 160 | 1536 | 320 |  | 320 | 1536 | 160 |  | 160 | 768 | 320 |  | 160 | 512 | 160 |
|  | 2074 |  | 320 | 3072 | 640 |  | 320 | 1536 | 640 |  | 160 | 768 | 320 |  | 160 | 1024 | 320 |
|  | 2075 |  | 160 | 1536 | 160 |  | 320 | 2048 | 320 |  | 80 | 384 | 40 |  | 160 | 1536 | 160 |
|  | 2076 |  | 160 | 1024 | 320 |  | 320 | 2048 | 640 |  | 80 | 192 | 160 |  | 160 | 1024 | 320 |
|  | **Mean** |  | **190** | **1651** | **320** |  | **320*** | **1774*** | **381** |  | **113** | **457** | **160** |  | **160** | **953** | **226** |

Data are shown at 2 (week 6) and 4 (week 8) weeks after the booster vaccination. HI: hemagglutination inhibition; VN: virus neutralization; NI: neuraminidase inhibition; n.t.: not tested. Geometric mean antibody titers are shown in bold. Asterisks denote significant differences (*P* < 0.05) between groups in the Mann-Whitney test.
