# Supplementary material for: Heterologous prime-boost vaccination with H3N2 influenza viruses of swine favors cross-clade antibody responses and protection
Source: NPJ Vaccines. 2017 Apr 20;2:11. doi: 10.1038/s41541-017-0012-x (PMC5604745; doi:10.1038/s41541-017-0012-x)
Supplement: Supplementary file 7 — Table S2 [file 41541_2017_12_MOESM7_ESM.docx]

**Table S2.** Tracheal and lung pathology after challenge of different vaccine groups with sw/Gent/172/2008 (G08) or sw/Pennsylvania/A01076777/2010 (PA10)

|  | Mean lesion score after challenge (no. of pigs with lesions) | | | | | | | | |
| --- | --- | --- | --- | --- | --- | --- | --- | --- | --- |
|  |  | G08 challenge | | |  |  | PA10 challenge | | |
|  |  | Macroscopic  pneumonia | Microscopic lesions | |  |  | Macroscopic  pneumonia | Microscopic lesions | |
| Group | *N* |  | Lung | Trachea |  | *N* |  | Lung | Trachea |
| Mock- vaccinated, challenged | 6 | 1,75% (2) | 5,08 (6) | 1,17 (5) |  | 6 | 2,00% (3) | 5,08 (6) | 1,33 (5) |
| Homologous prime-boost G08 | 5 | 0,20% (1) | 2,20 (5) | 0,20 (1) |  | 6 | 3,25% (6) | 6,42 (6) | 1,83 (6) |
| Homologous prime-boost PA10 | 6 | 2,17% (3) | 4,17 (6) | 1,83 (6) |  | 5 | 0% (0) | 3,80 (5) | 0,20 (1) |
| Heterologous prime-boost G08-PA10 | 7 | 0% (0) | 3,15 (7) | 0,29 (2) |  | 7 | 0,50% (2) | 3,93 (7) | 1,71 (6) |
| Bivalent vaccine G08+PA10, 1x  Bivalent vaccine G08+PA10, 2x | 6  2 | 0% (0)  0% (0) | 2,83 (6)  1,50 (2) | 0,83 (4)  0,00 (0) |  | 6  2 | 0,25% (1)  0% (0) | 3,67 (6)  2,50 (2) | 1,00 (3)  0,00 (0) |

Microscopic lung lesion scores are composite scores based on the severity of 3 parameters: (1) epithelial damage in intrapulmonary airways (0-3), (2) peribronchiolar lymphocytic cuffing (0-3), (3) neutrophil exudation in bronchioles and alveoli (0-2); Microscopic tracheal lesion scores are based on the severity of epithelial damage (0-2).

Unvaccinated, unchallenged control pigs (*n* = 4) had a mean macroscopic pneumonia score of 0, and mean microscopic lesion scores of 2 (lung) and 0 (trachea).
