# Supplementary material for: Heterologous prime-boost vaccination with H3N2 influenza viruses of swine favors cross-clade antibody responses and protection
Source: NPJ Vaccines. 2017 Apr 20;2:11. doi: 10.1038/s41541-017-0012-x (PMC5604745; doi:10.1038/s41541-017-0012-x)
Supplement: Supplementary file 8 — Table S3 [file 41541_2017_12_MOESM8_ESM.docx]

**Table S3.** HI and VN antibody titers against heterosubtypic influenza viruses after homologous and heterologous prime-boost vaccination

|  |  | No. of pigs with antibodies (titer range) | | | | | | |
| --- | --- | --- | --- | --- | --- | --- | --- | --- |
|  |  | HI | | |  | VN | | |
|  | 1° | G08 | PA10 | G08 |  | G08 | PA10 | G08 |
| Virus (subtype) | 2° | G08  *n* = 12 | PA10  *n* = 11 | PA10  *n* = 14 |  | G08  *n* = 12 | PA10  *n* = 11 | PA10  *n* = 14 |
| Sw/Gent/28/2010 (H1N1) |  | 0 | 0 | 0 |  | 0 | 0 | 0 |
| Sw/Gent/26/2012 (H1N2) |  | 0 | 0 | 2  (20-40) |  | 0 | 0 | 4  (16-24) |
| Mallard/Alberta/1998 (H4N1) |  | 0 | 0 | 1  (20) |  | 0 | 0 | 1  (96) |
| Duck/Belgium/06936/2005 (H4N6) |  | 0 | 0 | 1  (40) |  | 0 | 0 | 1  (192) |
| Chicken/Italy/1067/V1999 (H7N1) |  | 0 | 0 | 0 |  | 0 | 0 | 0 |

1°, 2°: Virus strains used for first and second vaccination; *n* = number of pigs examined
