# Supplementary material for: Heterologous prime-boost vaccination with H3N2 influenza viruses of swine favors cross-clade antibody responses and protection
Source: NPJ Vaccines. 2017 Apr 20;2:11. doi: 10.1038/s41541-017-0012-x (PMC5604745; doi:10.1038/s41541-017-0012-x)
Supplement: Supplementary file 9 — Supplementary Figure Legends [file 41541_2017_12_MOESM9_ESM.docx]

Fig. S1. Alignment of deduced amino acid sequences in the HA1 of the vaccine strain sw/Gent/172/2008 (G08) and other H3N2 viruses used in this study. Residues in the open boxes represent previously identified antigenic sites (A, B, C, D, and E) of H3. Only amino acids different from those in the G08 sequence are shown, conserved residues are shown as dots. Substitutions at positions 145, 155, 156, 158, 159, 189 and 193 are highlighted, because they have been associated with antigenic evolution in swine and in humans. Eleven of the 14 viruses aligned to G08 differ from the vaccine strain in at least 4 of these residues.

Fig. S2. Alignment of deduced amino acid sequences in the HA1 of the vaccine strain sw/Pennsylvania/A01076777/2010 (PA10) and other H3N2 viruses used in this study. Residues in the open boxes represent previously identified antigenic sites (A, B, C, D, and E) of H3. Only amino acids different from those in the PA10 sequence are shown, conserved residues are shown as dots. Substitutions at positions 145, 155, 156, 158, 159, 189 and 193 are highlighted, because they have been associated with antigenic evolution in swine and in humans. Eleven of the 14 viruses aligned to PA10 differ from the vaccine strain in at least 4 of these residues.

Fig. S3. Microscopic lung lesions in unvaccinated, unchallenged control pigs (*A*) and pigs challenged with PA10 (*B-F*). The extent of peribronchiolar lymphocytic cuffing and neutrophil infiltration was similar in the mock-vaccinated challenge control group (*D*) and the G08 homologous prime-boost group (*B*). Compared to the challenge control group milder lesions were observed in the PA10 homologous prime-boost group (*E*), the heterologous prime-boost group (*C*) and the bivalent vaccine (2x) group (*F*), but the differences were not statistically significant (*P* > 0.05, Kruskal-Wallis). None of the vaccine groups had enhanced lesions relative to the challenge control.

Fig S4. Comparison of HI antibody titers against heterologous H3N2 influenza viruses in pigs of different vaccine groups. Eu: European; NA: North American. Antibodies are shown 2 weeks after the booster vaccination (week 6). Titers of 40 (dotted line) are considered as seroprotective. Overall, sera from the heterologous prime-boost group were most cross-reactive.

Fig. S5. Minimal titers of anti-HA stalk antibodies in pigs of different vaccine groups. Stalk-reactive antibodies were measured in a cH5/3 ELISA. Sera from 4-6 pigs per group were examined at 2 weeks after the booster vaccination. Each symbol represents the titer of an individual pig, horizontal lines indicate group means. The dotted line indicates the limit of detection. Titers in the vaccinated groups did not differ from those in the mock-vaccinated control group (*P* > 0.05, Kruskal-Wallis).
